# Hexagonal CuCo_2_O_4_ Nanoplatelets, a Highly Active Catalyst for the Hydrolysis of Ammonia Borane for Hydrogen Production

**DOI:** 10.3390/nano9030360

**Published:** 2019-03-04

**Authors:** Jinyun Liao, Yufa Feng, Shiqi Wu, Huilong Ye, Jin Zhang, Xibin Zhang, Feiyan Xie, Hao Li

**Affiliations:** School of Chemistry and Materials Engineering, Huizhou University, Huizhou 516007, China; jyliao@126.com (J.L.); yufafeng@126.com (Y.F.); 15217835009@163.com (S.W.); yehuilong6364@163.com (H.Y.); eeedwardjin@163.com (J.Z.); zxbin1@163.com (X.Z.); xfy@hzu.edu.cn (F.X.)

**Keywords:** nanoplatelets, heterogeneous catalysis, hydrogen production, ammonia borane, viscosity

## Abstract

Catalytic hydrolysis of ammonia borane (AB) has been considered as an effective and safe method to generate hydrogen. Development of highly active and low-cost catalysts is one of the key tasks for this technology. In this work, hexagonal CuCo_2_O_4_ nanoplatelets with a thickness of approximately 55 nm were prepared. In AB hydrolysis, those nanoplatelets exhibited ultrahigh catalytic activity with turnover frequency (TOF) of 73.4 mol_hydrogen_ min^−1^ mol_cat_^−1^. As far as we know, this is one of the highest TOF values ever reported for non-noble metal catalysts. In addition, the effects of viscosity and different alkalis on the hydrolysis were also investigated. It is revealed that high viscosity of the reaction medium will retard the hydrolysis reaction. The presence of NaOH, KOH, and Na_2_CO_3_ in the reaction solution is favorable for hydrolytic process. In contrast, NH_3_·H_2_O will slow down the hydrolysis rate of ammonia borane. This work can provide some novel insight into the design of catalysts with both high performance and low cost. Besides, some findings in the present study can also offer us some information about how to improve the hydrolysis rates by optimizing the hydrolysis condition.

## 1. Introduction

Hydrolysis of ammonia borane (AB) is a very promising way to provide hydrogen for mobile hydrogen-oxygen fuel cells [1,2,3], which can find many important applications in new energy vehicles in the near future. According the reaction equation,

H_3_NBH_3_(aq) + 2H_2_O(l) → NH_4_^+^(aq) + BO_2_^−^(aq) + 3H_2_(g) (1)

per mole AB can produce 3 moles hydrogen by a simple hydrolytic reaction at room temperature and atmospheric pressure. In this hydrogen-production technology, a catalyst is necessary in view of the slow kinetic of the hydrolysis process. Since Xu’s pioneering works on AB hydrolysis catalyzed by transition metals was reported [4,5], a number of heterogeneous catalysts have been developed for that hydrolytic reaction [6,7,8,9,10,11]. Among these catalysts, noble metal based catalysts are too expensive despite their high catalytic performance [6,7,8]. In contrast, non-noble-metal catalysts are very cheap, but their activity is far from satisfactory [9,10,11]. Therefore, it is highly desirable to develop some catalysts with high catalytic performance and low cost, which is crucial for large-scale applications of this technology.

Generally speaking, there are three main routes to enhance the reaction rate of a catalytic process. The first one is development of a new type of catalyst with high catalytic activity. The second one is to increase the activity of the catalyst by surface decoration, foreign element doping, etc. The third one is to optimize the reaction conditions, such as temperature, pH value, viscosity, substrate/catalyst ratio. As for the third point, many parameters in the hydrolytic reaction of AB, such as reaction temperatures, catalyst dosages, substrate concentrations, have been extensively investigated in previous works [12,13,14,15,16,17,18]. However, other factors—including viscosity of the medium, alkali—have been seldom concerned, which may significantly affect the hydrolysis rate of AB. As for the effect of alkali on AB hydrolysis, we have found in our previous work that a proper amount of NaOH in the reaction medium can significantly enhance AB hydrolysis [9]. However, the effects of the concentration of the alkali on the rate of AB hydrolysis have not been systematically investigated. Thus, whether there is an optimal concentration of alkali for AB hydrolysis remains unclear. In the practical application of AB hydrolysis, many factors—such as temperature, additive in solution, and the byproduct of the reaction—may affect the viscosity of the reaction medium, which will affect the reaction rate of AB hydrolysis. Thus, it is of importance for us to know the impact of the viscosity to hydrolysis reaction.

In this work, hexagonal CuCo_2_O_4_ nanoplatelets with a thickness of about 55 nm were prepared by a hydrothermal approach followed by a calcination process. Vijayakumar et al. have prepared CuCoO4 nanobelts by using Co(NO_3_)_2_/Cu(NO_3_)_2_ as Co/Cu resource, urea as precipitant, and sodium dodecyl sulfate (SDS) as surfactant, respectively [19]. Jadhav et al. have grown the flower-like CuCo_2_O_4_ on Ni foam by hydrothermal method followed by heat treatment [20]. Sun et al. have reported the synthesis of 3D free-standing hierarchical CuCo_2_O_4_ nanowires by a hydrothermal method [21]. As far as we know, CuCo_2_O_4_ nanoplatelets have not been reported yet in the literature. In AB hydrolysis, those nanoplatelets exhibited ultrahigh catalytic activity with turnover frequency (TOF) of 73.4 mol_hydrogen_ min^−1^ mol_cat_^−1^, which is higher than those of CoP nanoparticles (72.7 mol_hydrogen_ min^−1^ mol_cat_^−1^) [22], Cu_0.8_Co_0.2_O/Graphene oxide (70.0 mol_hydrogen_ min^−1^ mol_cat_^−1^) [23], Ni_0.9_Mo_0.1_/graphene (66.7 mol_hydrogen_ min^−1^ mol_cat_^−1^) [24], CuCo/diamine-functionalized reduced graphene oxide 51.5 mol_hydrogen_ min^−1^ mol_cat_^−1^) [25], NiCo_2_O_4_/Ti (50.1 mol_hydrogen_ min^−1^ mol_cat_^−1^) [12], CoP nanoarray (42.8 mol_hydrogen_ min^−1^ mol_cat_^−1^) [26], Co/CTF(42.3 mol_hydrogen_ min^−1^ mol_cat_^−1^) [27], Ni nanoparticles supported on three-dimensional N-doped graphene (41.7 mol_hydrogen_ min^−1^ mol_cat_^−1^) [28], CuCo@MIL-101(19.6 mol_hydrogen_ min^−1^ mol_cat_^−1^) [29], and CoNi/Graphene (16.8 mol_hydrogen_ min^−1^ mol_cat_^−1^) [30], However, this TOF value is still lower than that of Cu_0.6_Ni_0.4_Co_2_O_4_ nanowires (119.5 mol_hydrogen_ min^−1^ mol_cat_^−1^) [2], Ni/ZIF-8 nanocatalyst (85.7 mol_hydrogen_ min^−1^ mol_cat_^−1^) [31], and CuCo/g-C_3_N_4_-1(75.1 mol_hydrogen_ min^−1^ mol_cat_^−1^) [32]. In the viscosity range from 1.08 to 32.95 mPa·s, high viscosity has a negative effect on AB hydrolysis. In addition, it is revealed that NaOH and Na_2_CO_3_ can enhance AB hydrolysis but NH_3_·H_2_O will retard that hydrolytic reaction.

## 2. Experimental Section

In a typical process, 4.0 mmol Co(CH_3_COO)_2_ and 2.0 mmol CuSO_4_ were mixed in 20 mL water by stirring. Subsequently, 20 mol ethanolamine was added to a beaker containing 20 mL water. After the two solutions were blended, 40 mL NaOH solution (2.5 M) was added slowly into the mixture. The obtained suspension was transferred into a Teflon-lined stainless autoclave, which was sealed and placed into a drying oven. The temperature was maintained at 160 °C for 8 h. When the hydrothermal treatment finished, the resultant solid products were collected, rinsed and subjected to a heat treatment of 600 °C for 2 h. The heating rate was 10 °C/min and the calcination was carried out in the air.

Rigaku TTR3 X-ray powder diffractometer (Tokyo, Japan) with a Cu K radiation (λ = 1.5406 Å) was applied to record the powder X-ray diffraction (XRD) patterns of the samples. Hitachi SU-8100 scanning electron microscope (Hitachi, Japan) was utilized to observe the morphology of the catalysts. Tecnai G2 F20 S-TWINT transmission electron microscope (FEI, Hillsboro, OR, USA) was used to obtain TEM and HRTEM images. Kratos Axis Ultra (DLD) X-ray photoelectron spectroscope was applied to analyze the elements and chemical states on the surface of the sample. Viscosity was determined with a NDJ-5S Digital Viscometer (Shanghai Lichen Bangxi Instrument Co. Ltd., Shanghai, China).

Typically, 10.0 mg catalyst (ca. 0.0435 mmol) was dispersed 5.0 mL water by ultrasonication treatment in the reaction vessel, which was then put into a water bath for maintaining the reaction temperature of 298 K. After that, 15 mL mixture solution containing NaOH (20.0 mmol) and AB (2.6 mmol) was added into the vessel, which was connected to a graduated gas burette. The volume of produced hydrogen was determined by water displacement in the burette. In order to investigate that effect of viscosity of solution on the release rate of hydrogen, a glycerol/water mixture instead of water was used a solution, and viscosity was measured at 298 K.

## 3. Results and Discussion

**Figure 1:**
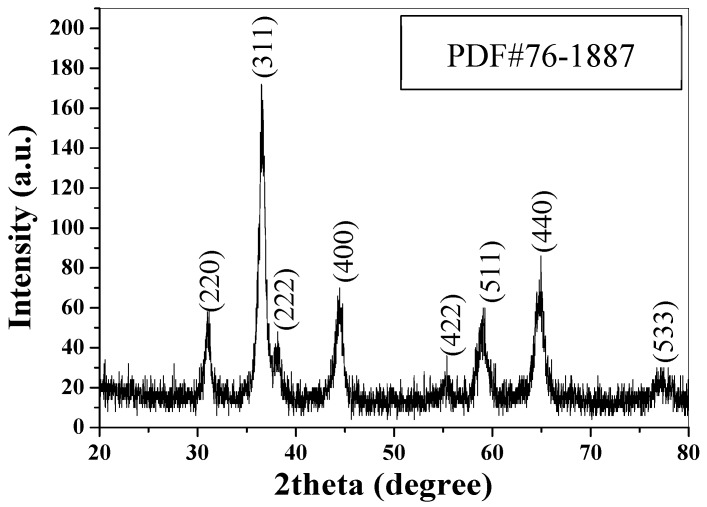
XRD pattern of the CuCo_2_O_4_ nanoplatelets.

Figure 1 shows the XRD pattern of the as-prepared CuCo_2_O_4_ nanoplatelets. As shown, eight characteristic peaks at 2*θ* = 31.1°, 36.6°, 38.3°, 44.5°, 55.5°, 59.0°, 65.0°, and 77.1° are observed, corresponding to the diffraction from (220), (311), (222), (400), (422), (511), (440), and (533) planes of spinel phase of CuCo_2_O_4_ (PDF#76-1887). According to the Scherrer equation, the crystallite size is calculated to be 16.6 nm based on the (311) peak width at half-height, which is the peak with highest intensity. In the basic reaction medium, Co^2+^ and Cu^2+^ ions will be changed into Co(OH)_2_ and Cu(OH)_2_, respectively. During the calcination process, CuCo_2_O_4_ is formed. The reaction can be formulated as

2Co^2+^ + Cu^2+^ + 6OH^−^ → 2Co(OH)_2_ + Cu(OH)_2_ (2)

2Co(OH)_2_ + Cu(OH)_2_ + 1/2O_2_ → CuCo_2_O_4_ + 3H_2_O (3)

**Figure 2:**
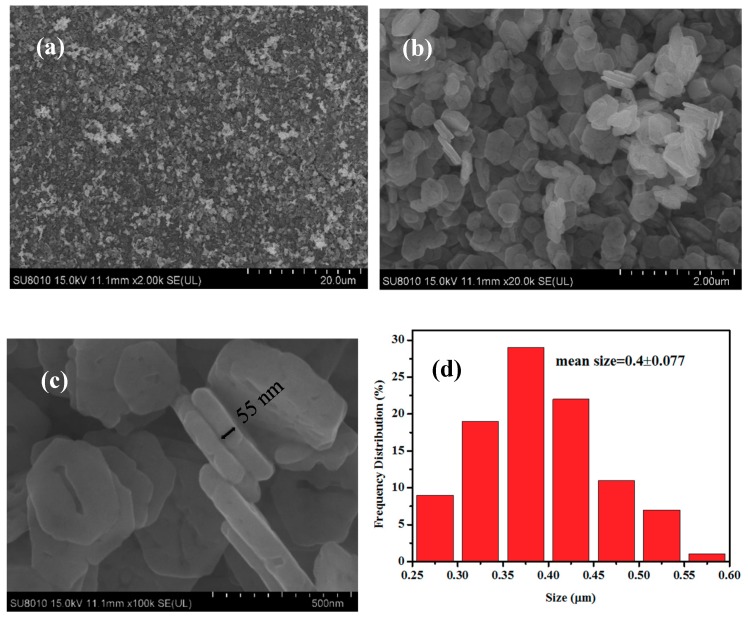
SEM images (**a**–**c**) and the size distribution (**d**) of the CuCo_2_O_4_ nanoplatelets.

**Figure 3:**
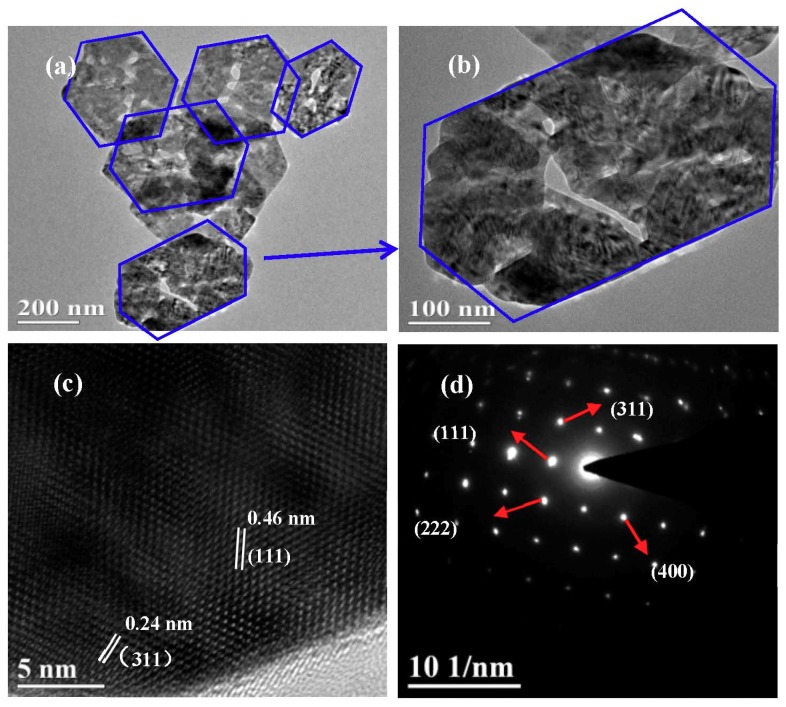
TEM images (**a**,**b**), HRTEM image (**c**) and SAED pattern (**d**) of the CuCo_2_O_4_ nanoplatelets.

Figure 2a,b are the low-magnification SEM images of the CuCo_2_O_4_ samples, indicating that a large amount of CuCo_2_O_4_ nanoplatelets can be obtained by our synthetic route. Figure 2c shows that the typical thickness and size of the hexagonal nanoplatelets are around 55 nm. Particle size distribution in Figure 2d indicates that the mean size of the nanoplatelets is around 400 nm. Figure 3a,b are the TEM images of the CuCo_2_O_4_ sample, which further confirms the hexagonal structure of nanoplatelets. HRTEM image in Figure 3c displays the crystal distance of 0.46, 0.24 nm, corresponding to the (111) and (311) planes of spinel phase of CuCo_2_O_4_ [33,34,35]. The selected area electron diffraction (SAED) pattern demonstrates that the CuCo_2_O_4_ nanoplatelets are well crystalized.

To know more about the formation of CuCo_2_O_4_ nanoplatelets, we carried out some control experiments. First, we synthesized the CuCo_2_O_4_ sample in the absence of ethanolamine. SEM image (Figure S1a) indicates the product is the aggregation composed of irregularly shaped nanoparticles. When the sodium citrate instead of ethanolamine is used as complexing agent, nanoparticles and nanoplatelets coexist in the sample (Figure S1b). These observations hint that ethanolamine plays an important role in determining the final morphology of the sample.

**Figure 4:**
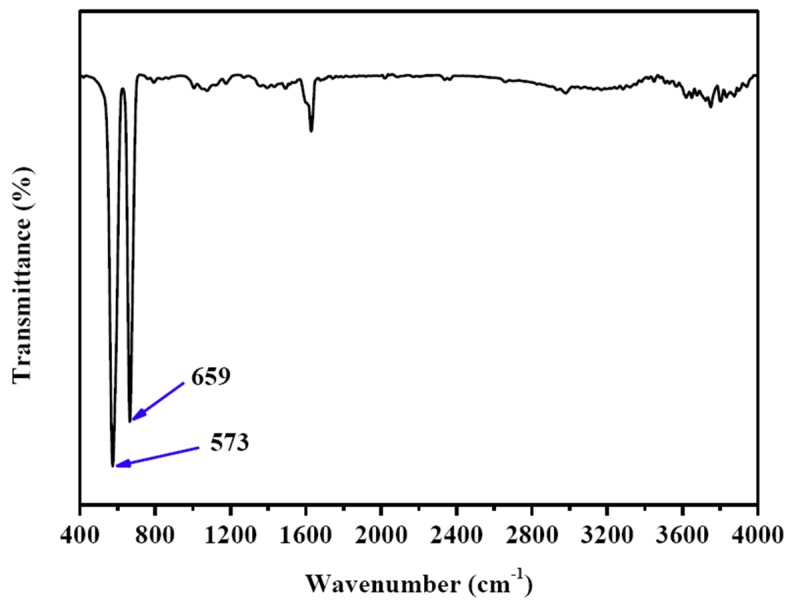
FTIR spectrum of the CuCo_2_O_4_ nanoplatelets.

Figure 4 is the FTIR spectrum of CuCo_2_O_4_ nanoplatelets, in which two peaks at 573 and 659 cm^−1^ can be observed. These two peaks can be indexed to the Co^3+^-O^2−^ and Cu^2+^-O^2−^ functional groups in the CuCo_2_O_4_, further demonstrating that our sample is CuCo_2_O_4_ [36].

**Figure 5:**
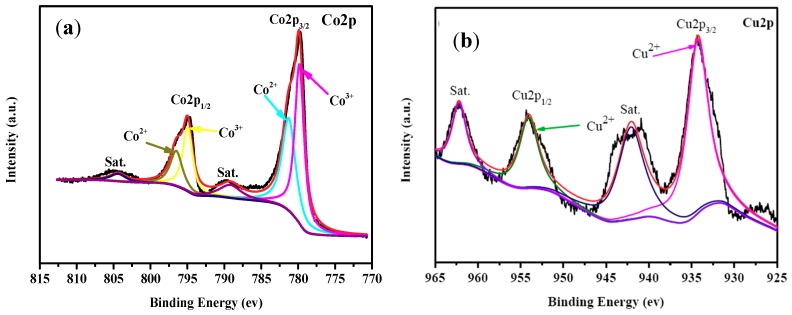
XPS spectra of the CuCo_2_O_4_ nanoplatelets in Co2p (**a**) and Cu2p (**b**) regions.

The elements on the surface of CuCo_2_O_4_ nanoplatelets were analyzed with XPS and the results shown in Figure 5. Figure 5a is the XPS spectrum of Co2p of the CuCo_2_O_4_ nanoplatelets. As it can be seen in Figure 5a, the Co (2p_1/2_) peak can be decomposed into two peaks at 796.6 eV and 794.8 eV. Meanwhile, the Co (2p_3/2_) peak can be decomposed into two peaks at 781.3 eV and 779.8 eV. The peaks at 796.6 eV and 781.3 eV are attributed to the Co^2+^ state and those at of 794.8 eV and 779.8 eV are indexed to the Co^3+^ state [19]. It should be mentioned that the spin-orbit splitting for the Co^2+^ doublet is 15.3 eV and for the Co^3+^ doublet is 15.0 eV, hinting that the these Co species are presented as cobalt oxides instead of cobalt hydroxides [37]. Similarly, two peaks at 954.3 eV and 934.2 eV are observable in the Cu 2p region of the XPS spectra (Figure 5b), which is related to the Cu (2p_1/2_) and Cu (2p_3/2_) peaks, respectively [19]. Besides, two peaks at 962.3 eV and 942.2 eV can be seen, which are the satellite peaks of Cu 2p. All these peak values are in good agreement with those for Cu^2+^ (CuCo_2_O_4_) in previous works [19,38]. In summary, Cu^2+^, Co^2+^, and Co^3+^ have been detected with XPS on the surface of the CuCo_2_O_4_ sample. This observation is coincident with those XPS results reported for CuCo_2_O_4_ in the literature.

**Figure 6:**
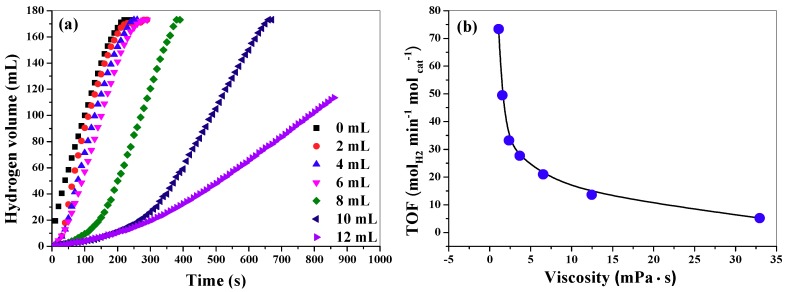
Hydrogen release curves in mixed solvent with different volume of glycerol. The total volume of water and glycerol before mixing is fixed at 20 mL.

Viscosity is a very important parameter in the reaction medium, which may significantly affect the reaction rate. However, to the best of our knowledge, the effect of viscosity on the hydrolysis of AB has not been reported yet. In this work, for the purpose of investigating viscosity effect, 0–12 mL glycerol is introduced into the reaction medium. It should be mentioned that the total volume of water and glycerol before mixing is fixed at 20 mL. Note that the 2.6 mmol AB will react with 5.2 mmol water (0.094 mL). Thus, the water in the reaction medium is remarkably excessive even in the case of only 8 mL water in the mixed solvent. On the other aspect, although AB can react with methanol to produce hydrogen by a catalytic methanolysis process, we found that nearly no hydrogen was produced when AB/glycerol was solvent and CuCo_2_O_4_ nanoplatelets acted as a catalyst in a preliminary experiment. This indicates that glycerol can hardly react with AB, but it can adjust the viscosity of the reaction medium. Therefore, the effect of glycerol on the hydrolysis can be attributed to the viscosity. As shown in Figure 6a, the hydrogen generation rate decreases when the volume of glycerol increases. In addition, the induction time of the hydrolysis reaction will increase as the increase of the volume of glycerol. For example, when the volume of glycerol is no more than 4 mL, it will take less than 10 s to produce hydrogen after the catalyst comes into contact with the reaction solution. In contrast, when the volume of glycerol in reaction medium increases to 12 mL, the induction time reaches about 60 s. Table 1 shows the data of viscosity of reaction medium, the corresponding TOF values and induction time. Figure 6b shows the relationship between the TOF values and the viscosity. According to the mechanism for AB hydrolysis proposed by Mahyari et al., the transportation of the AB molecule to the catalyst surface to form an activated complex species are the key step in AB hydrolysis [28]. Thus, the AB transportation rate and the desorption rate of the intermediates from catalyst surface will influence the overall hydrolysis rate. Evidently, high viscosity will lower the mass transportation rate and therefore the TOF values decrease and meantime the induction time increases.

To exclude the possibility of the activity loss of the catalyst caused by surface poisoning via glycerol adsorption, we carried out a series of contrast experiments, in which the viscosity of the reaction medium was adjusted with ethylene glycol instead of glycerol. The total volume of water and ethylene glycol before mixing is still fixed at 20 mL. When the volume of ethylene glycol is 2, 6, and 12 mL, the viscosity of the reaction medium is 1.52, 2.83, and 8.33, and the corresponding TOF values are 48.7, 31.1, and 15.6 mol_H2_ min^−1^ mol_cat_^−1^, respectively. Additionally, we also adjusted the viscosity of reaction medium by polyacryamide. It is found that when the viscosity of the reaction medium is 3.25, 4.3, 6.4, and 9.3 mPa·s, and the corresponding TOF values are 12.1, 9.3, 8.8, and 7.1 mol_H2_ min^−1^ mol_cat_^−1^, respectively. These observations further imply that it is the high viscosity of reaction medium that cause the activity decrease.

**Figure 7:**
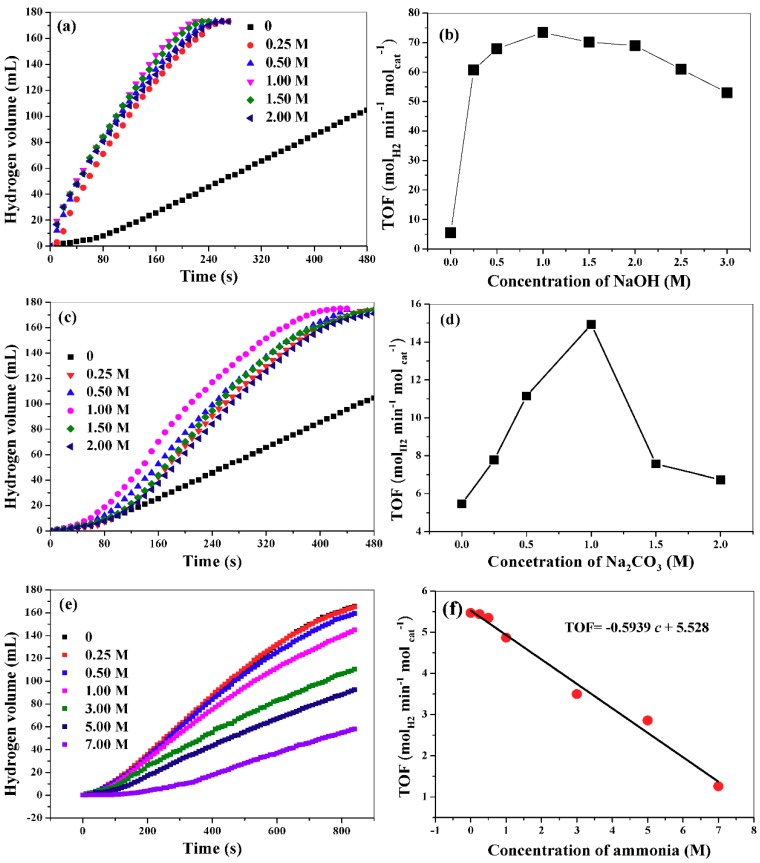
Hydrogen release curves in the presence of NaOH (**a**), Na_2_CO_3_ (**c**), and NH_3_·H_2_O (**e**), and the corresponding TOF values at different concentrations (**b**,**d**,**f**).

In this work, we have investigated the influence of NaOH, Na_2_CO_3_ and ammonia on AB hydrolysis. Figure 7a,b show the hydrogen release curves from reaction medium with different NaOH concentration and the corresponding TOF values. Evidently, the introduction of NaOH will remarkably affect the rate of hydrogen production. In the absence of NaOH in the reaction medium, the TOF is only 5.47 mol_hydrogen_ min^−1^ mol_cat_^−1^. In contrast, the TOF increase to 73.4 mol_hydrogen_ min^−1^ mol_cat_^−1^ at NaOH concentration of 1.0 M. A further increase of NaOH concentration will result in a slight decrease of TOF value. According to reaction (1), NH_4_^+^ will be formed during the hydrolytic process. When OH^−^ is added into the reaction medium, it will react with NH_4_^+^ by the following reaction. NH_4_^+^ + OH^−^ → NH_3_·H_2_O. Thus, from the viewpoint of shift of chemical equilibrium, the hydrolysis reaction may be enhanced by introducing OH^−^ into the reaction system. In addition, the introduction of OH^−^ into the reaction system can improve the electronic properties of the catalyst, which is favorable for the interaction with catalyst and AB [18]. However, excessive NaOH (>1 M) has a negative effect on AB hydrolysis. We find that the viscosity of the reaction medium increases as NaOH concentration increases (please see the relationship between the viscosity and NaOH concentrations in Figure S2), which will lead to the slow transportation of AB from bulk solution to the surface of the catalyst. Therefore, the hydrolytic process is hindered and the reaction rates decrease. We also investigate the effect of KOH concentration on AB hydrolysis and find that KOH concentration has almost the same influence on AB hydrolysis as NaOH. This implies that it is the anions (OH^-^) instead of the cations (Na^+^ or K^+^) that affect the hydrolysis reaction. Figure 7c,d show the hydrogen release curve in the presence of Na_2_CO_3_ with different concentrations and the corresponding TOF values variation. Although Na_2_CO_3_ can also improve the hydrolysis of AB, its impact is significantly lower than those of NaOH and KOH. This is understandable because Na_2_CO_3_ is a weak alkali and can provide less OH^-^ than NaOH and KOH. Note that ammonia gas may be generated in basic solution, which will contaminate the collected hydrogen. However, the obtained gas could be easily depurated with water or an acidic solution, which will absorb ammonia gas. As for the effect of ammonia on AB hydrolysis, the TOF value will decrease when the concentration of ammonia is larger than 0.25 M (Figure 7e). As shown in Figure 7f, TOF will decrease linearly with the increase of the concentration of ammonia. Quite different with the effects of other alkalis on AB hydrolysis, NH_3_·H_2_O shows negative influence on the hydrolytic reaction of AB. NH_3_·H_2_O will produce NH_4_^+^ via a dissociation reaction, NH_3_·H_2_O → NH_4_^+^ + OH^−^, which may retard AB hydrolysis.

**Figure 8:**
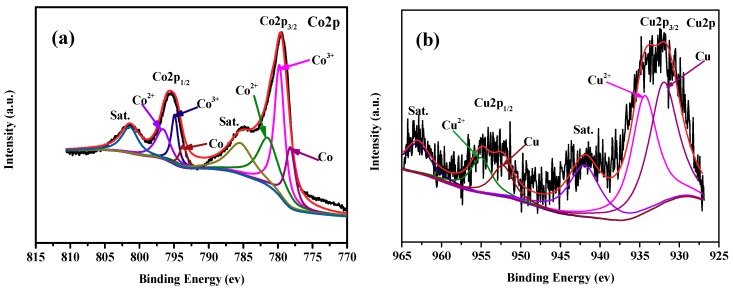
XPS specta of the CuCo_2_O_4_ catalyst in Co2p (**a**) and Cu2p (**b**) regions after catalytic reaction.

In our previous work, we have found that the active species of Cu_0.6_Ni_0.4_Co_2_O_4_ in AB hydrolysis is metallic Co, Ni, and Cu on the catalyst surface, which were formed by the reduction of Cu_0.6_Ni_0.4_Co_2_O_4_ with AB [2]. In the present study, it is highly believed that metallic Co and Cu on the catalyst surface, which were formed by the similar process, can serve as the active species. Actually, XPS data of the used catalyst (Figure 8) demonstrates that the formation of metallic Co and Cu on the surface of the catalyst. The synergistic effect of resultant metallic Co and Cu on CuCo_2_O_4_ surface can be attributed to the relatively high catalytic activity of our catalyst in AB hydrolysis. In addition, metallic Co and Cu on the catalyst surface, and the internal CuCo_2_O_4_ can form a metal−support interaction, which is favorable for AB hydrolysis [1]. This may be another reason for the high activity of our CuCo_2_O_4_ catalyst. SEM image of the used catalyst (Figure S3) indicates that the morphology of the nanoplatelets is still retained after reaction.

## 4. Conclusions

In summary, CuCo_2_O_4_ nanoplatelets with a thickness of about 55 nm were prepared by a hydrothermal approach followed by a calcination process. In AB hydrolysis, those nanoplatelets exhibited superior catalytic activity with TOF of 73.4 mol_hydrogen_ min^−1^ mol_cat_^−1^. To know the optimal conditions for AB hydrolysis catalyzed by CuCo_2_O_4_ nanoplatelets, we also investigated the effects of alkali with different concentration and viscosity on the hydrolytic reaction. It was revealed that both NaOH, and Na_2_CO_3_ in the reaction medium could improve the hydrolysis rate of AB. In particular, the TOF value in the presence of a suitable concentration of NaOH is more than 10 times as high as that in the absence of NaOH. In contrast, ammonia would retard the hydrolytic reaction. In addition, high viscosity of reaction medium had a significant negative effect on AB hydrolysis. These observations are helpful for us to select the proper reaction conditions for AB hydrolysis in practical applications.

## Figures and Tables

**Table 1:** Data of viscosity of reaction medium and the corresponding TOF values and induction times.

Volume of Glycerol in Reaction Medium (mL)	Viscosity (mPa·s)	TOF (mol_H2_ min^−1^ mol_cat_^−1^)	Induction Time(s)
0	1.08	73.4	2
2	1.54	49.5	8
4	2.35	33.2	8
6	3.67	27.7	20
8	6.52	21.0	35
10	12.45	13.6	47
12	32.95	5.2	56

## Supplementary Materials

The following are available online at https://www.mdpi.com/2079-4991/9/3/360/s1, Figure S1: SEM images of the sample prepared in the absence of ethanolamine and the sample prepared by using sodium citrate instead of ethanolamine as complexing agent, Figure S2: Effect of NaOH concentrations on the viscosity of the medium, Figure S3: SEM image of the CuCo_2_O_4_ nanoplatelets after catalytic reaction.
