# Supplementary material for: Hexagonal CuCo2O4 Nanoplatelets, a Highly Active Catalyst for the Hydrolysis of Ammonia Borane for Hydrogen Production
Source: Nanomaterials (Basel). 2019 Mar 4;9(3):360. doi: 10.3390/nano9030360 (PMC6473973; doi:10.3390/nano9030360)
Supplement: Supplementary file 1 [file nanomaterials-09-00360-s001.pdf]

Supplementary Materials

Hexagonal $CuCo_2O_4$ Nanoplatelets, a Highly Active Catalyst for the Hydrolysis of Ammonia Borane for Hydrogen Production

Jinyun Liao, Yufa Feng, Shiqi Wu, Huilong Ye, Jin Zhang, Xibin Zhang, Feiyan Xie, and Hao Li *

School of Chemistry and Materials Engineering, Huizhou University, Huizhou 516007, China; jyliao@126.com (J.L.); yufafeng@126.com (Y.F.); 15217835009@163.com (S.W.); yehuilong6364@163.com (H.Y.); eeedwardjin@163.com (J.Z.); zxbin1@163.com (X.Z.); xfy@hzu.edu.cn (F.X.)

* Correspondence:Correspondence: lihao180@126.com; Tel.: +86-752-252-7229

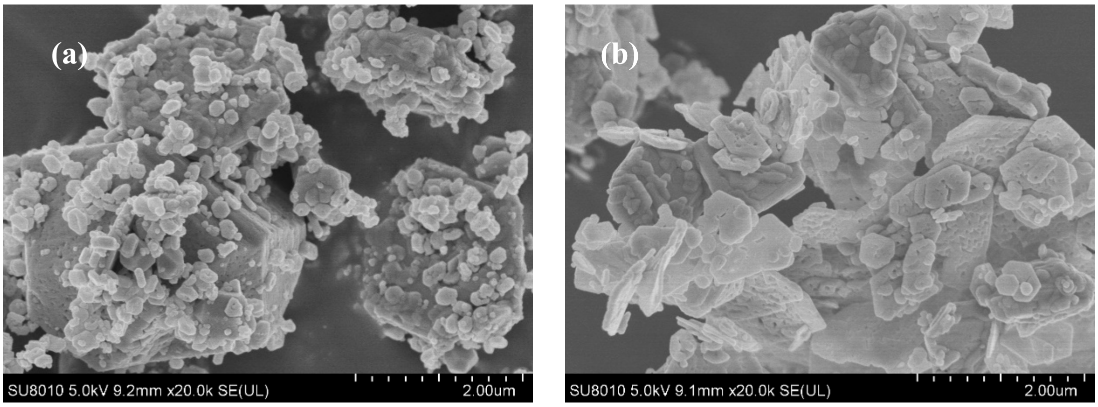

Figure S1 SEM images of the sample prepared in the absence of ethanolamine (a) and the sample prepared by using sodium citrate instead of ethanolamine as complexing agent (b).

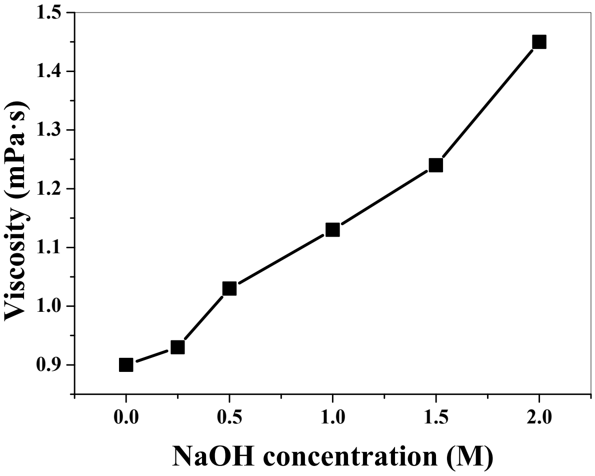

Figure S2: Effect of NaOH concentrations on the viscosity of the medium.

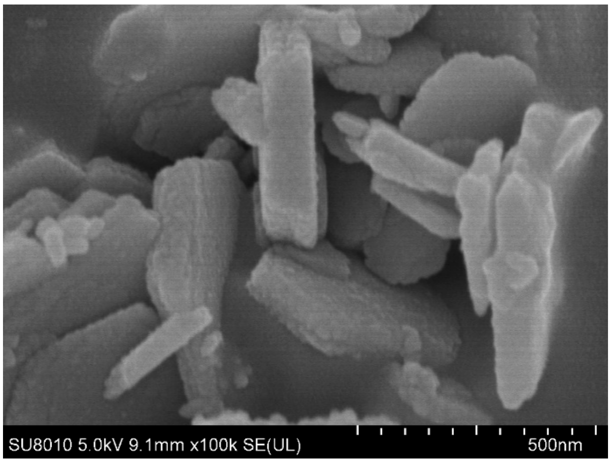

Figure S3: SEM image of the $CuCo_2O_4$ nanoplatelets after catalytic reaction.
